# Effectiveness of SGA-LAIs on Clinical, Cognitive, and Social Domains in Schizophrenia: Results from a Prospective Naturalistic Study

**DOI:** 10.3390/brainsci13040577

**Published:** 2023-03-29

**Authors:** Renato de Filippis, Filippo Antonio Staltari, Matteo Aloi, Elvira Anna Carbone, Marianna Rania, Laura Destefano, Luca Steardo Jr., Cristina Segura-Garcia, Pasquale De Fazio

**Affiliations:** 1 Psychiatry Unit, Department of Health Sciences, University Magna Graecia of Catanzaro, 88100 Catanzaro, Italy; 2 Department of Clinical and Experimental Medicine, University of Messina, 98125 Messina, Italy; 3 Psychiatry Unit, Department of Medical and Surgical Sciences, University Magna Graecia of Catanzaro, 88100 Catanzaro, Italy; 4 Center for Clinical Research and Treatment of Eating Disorders, University Hospital Mater Domini, 88100 Catanzaro, Italy

**Keywords:** antipsychotics, aripiprazole monohydrate, clinical study, executive functions, long-acting injectable, paliperidone palmitate, psychopharmacology, psychosis, schizophrenia, treatment

## Abstract

We hypothesized that shifting from oral second-generation antipsychotics (SGA) to their long-acting injectable (LAI) counterpart would be beneficial for the psychopathological, cognitive, social, and general health domains in outpatients suffering from schizophrenia. We aimed to evaluate the prospective usefulness of SGA-LAI treatment by carrying out a head-to-head comparison of two different medications (i.e., aripiprazole monohydrate (Ari-LAI) and paliperidone palmitate 1 and 3 month (PP1M, PP3M)) in a real-world setting, assessing the effectiveness and tolerability of Ari-LAI and PP1M/PP3M over a 15 month follow-up. A total of 69 consecutive individuals affected by schizophrenia were screened for eligibility. Finally, 46 outpatients (29 treated with Ari-LAI, 13 with PP1M, and four with PP3M) were evaluated through clinical, functional, and neuropsychological assessment administrated at baseline and after 3-, 12-, and 15-month follow-up periods. Moreover, periodic general medical evaluations were carried out. We estimated an overall improvement over time on the explored outcomes, without differences with respect to the type of LAI investigated, and with a global 16.4% dropout rate. Our findings suggest that switching from oral SGA to SGA-LAIs represents a valid and effective treatment strategy, with significant improvements on psychopathological, cognitive, social, and clinical variables for patients suffering from schizophrenia, regardless of the type of molecule chosen.

## 1. Introduction

Over the last four decades, a significant amount of public, private, academic, and industrial resources have been devoted to research and development of new treatments for severe psychiatric disorders (e.g., bipolar disorder, major depressive disorder and schizophrenia spectrum disorders—BD, MDD, SSDs), and mental health wellbeing in the general population [1]. In recent years, there has been growing interest in the development and approval of new antipsychotic (APs) drugs, and long-acting injectables (LAIs) formulations for already registered APs (LAI-APs) [2]. On this regard, LAI-APs first appeared in the late 1960s with the main goal of improving treatment adherence and decreasing discontinuation rates in patients who were poorly adherent but presented a satisfying clinical response to oral AP treatments [3]. Although issues of treatment non-adherence, partial adherence, and discontinuation have somewhat persisted over time [4], more recently, the opportunity to reduce the daily burden of oral formulations and the role of shared decision making become central topics in the LAI-APs prescription debate [5,6], even for patients suffering from first-episode psychosis (FEP) [7], elderly populations [8], those with co-occurring drug abuse disorder [9], and in the long-term follow-up period [10].

In view of that, usually, both first- (FGA) and second-generation (SGA) LAI-APs are mainly investigated for their effectiveness and tolerability profiles [11,12,13], while the impact on neurocognition, social cognition, and general health in everyday clinical practice is less investigated but highly desirable [14,15].

In this scenario, considering the increasing number of contexts, indications, and patient populations potentially eligible for LAI-AP treatment, more comprehensive real-world prospective evaluations of the effectiveness of LAI-AP therapy on psychopathological, cognitive, social, and clinical variables are strongly needed. Hence, we aimed to expand the sample and update the follow-up observation time of our previous work about the role of SGA-LAIs in schizophrenia (SCZ) management [16].

We hypothesize that shifting from the oral SGA formulation to their LAI counterpart should be beneficial for psychopathological, cognitive, social, and clinical domains.

Therefore, this clinical research study aims to evaluate the prospective effectiveness of SGA-LAI treatment in a sample of patients suffering from SCZ, providing a clinical evaluation potentially tackling the challenging task to selecting the optimal treatment that will provide an adequate balance between effectiveness, safety, tolerability, and adherence to patients.

Our second aim is to carry out a head-to-head comparison of two different SGA-LAI treatments (i.e., Ari-LAI, PP1M/PP3M) in an independent and real-world setting.

## 2. Materials and Methods

Participants were consecutively recruited at the Psychiatry Unit of the University Hospital Magna Graecia of Catanzaro (Italy). They were deemed eligible if they fulfilled the following criteria: (1) aged between 18 and 65 years and able to provide the informed consent form; (2) diagnosed with SCZ by a senior psychiatrist according to the DSM-5 diagnostic criteria; (3) stabilized with oral SGA therapy also available in LAI formulation (i.e., aripiprazole, paliperidone) for at least 6 months before enrollment in the study; (4) free from substance abuse disorder and dependence for 6 and 12 months, respectively. On the other hand, approached participants were excluded in case of: (1) recent (i.e., below 12 months) or uncertain diagnosis of SCZ; (2) presence of any other mayor psychiatric disorder according to the DSM-5 diagnostic criteria; (3) medical history that was undocumented; (4) history of a medical or neurological disorders that could affect cognitive function.

**Figure 1:**
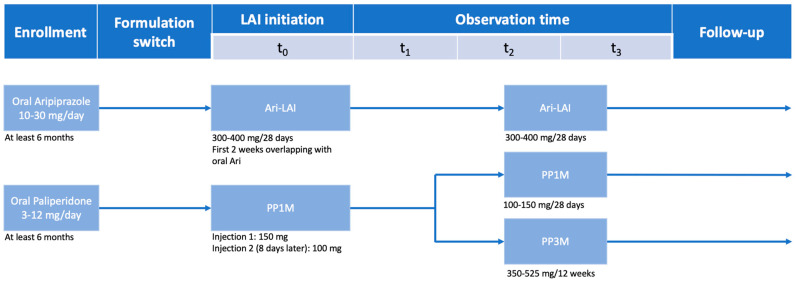
Study design infographics. Ari-LAI: LAI aripiprazole; PP1M: paliperidone palmitate 1 month; PP3M: paliperidone palmitate 3 months; t_0_: baseline; t_1_: 3 months; t_2_: 12 months; t_3_: 15 months.

Participants received either aripiprazole or paliperidone treatment, first as an oral formulation and then as LAI every 1 or 3 months (i.e., Ari-LAI, PP1M, PP3M), according to clinical judgment and without any study influence. Patients were switched to PP3M after 12 consecutive months of PP1M treatment with a satisfying response. The dosage range for oral formulations varied between 10 and 30 mg/day for aripiprazole and 3–12 mg/day for paliperidone, while the LAI formulations ranged for Ari-LAI, PP1M and PP3m, respectively, 300–400 mg/28 days, 100–150 mg/28 days, and 350–525 mg/12 weeks, according to good clinical practice evaluation (Figure 1).

It should be noted that in this paper, we refer to doses of PP1M and PP3M in terms of mg equivalents (mg eq.) of paliperidone (i.e., 25, 50, 75, 100, and 150 mg eq. for PP1M, and 175, 263, 350, and 525 mg eq. for PP3M), which correspond to doses of paliperidone palmitate substance 39, 78, 117, 156, and 234 mg, and 273, 410, 546 and 819 mg, respectively, with a conversion factor from mg eq. to mg of 1.56 [17].

The study followed all participants for a period of 15 months, and data collection took place between July 2016 and October 2020, with the final participant completing their follow-up and assessment. Data were gathered at four different time points: baseline (t_0_), 3 months (t_1_), 12 months (t_2_), and 15 months (t_3_), as shown in Figure 1 [16]. Before any procedure were carried out, the participants were given a thorough explanation of the study’s objectives and methods and provided their informed consent for participation. Participants were duly notified of their right to withdraw from the study at any point and were guaranteed to be carefully monitored for a safe discontinuation process so that they would not lose any benefits.

Before collecting any data, the study protocol was submitted to and approved by the local Ethical Committee of University Hospital Mater Domini of Catanzaro (Italy), ‘Regione Calabria, sezione Area Centro’. The study procedures and protocol were in accordance with the ethical principles outlined in the updated version of the Helsinki Declaration [18].

### 2.1. Assessment

As per the protocol, all participants underwent periodic clinical follow-up visits where they were assessed using specific psychometric tests administered by experienced clinicians during each visit.

#### 2.1.1. Psychopathological Assessment

- Positive and Negative Syndrome Scale (PANSS) [19]: It is a rater-administrated scale that aims to evaluate the occurrence and severity of positive and negative general psychotic symptoms, and disorganization of thought and behavior. The tool counts 30 items, assessable with a value from 1 to 7, theoretically divided into three sub-scales: seven-item scales for positive and negative symptoms, and a 16 item scale covering general psychopathology.

#### 2.1.2. Functional Assessment

- Quality of life scale (QoLS) [20]: It is a 16 item self-administered tool that allows the assessment of the self-perceived quality of life according to five conceptual domains: psychic and physical well-being, interpersonal relationships, civic and community activities, personal development, and recreational activities. Finally, the sum of individual items scores leads to a total score.
- Personal and Social Performance Scale (PSP) [21]: It is a rater-administrated tool scale that evaluates several dimensions of the patient’s overall personal functioning, assigning scores ranging between 1 and 6 to four domains: study and work activities, interpersonal relationships, self-care, aggression, and behavioral disorders. While the global functioning level is defined by a score ranging from a minimum of 1 to a maximum of 100.

#### 2.1.3. Neuropsychological Assessment

- Stroop Color and Word Test (SCWT) [22]: It is a cognitive test that evaluates the subject’s ability to inhibit cognitive interference, interference control, verbal speed, flexibility, and attention. The cognitive interference is measured through the increase response latencies and/or errors (Stroop effect) in colors recognition even with confounding factors, and these changes provide an index of cognitive flexibility.
- Rey-Osterrieth Complex Figure Test (RCFT) [23]: It is a rater-administrated tool that assesses the individual’s short-term visual memory, visual organization, and visuospatial abilities. Additionally, the RCFT can be used to examine organizational strategies used during the copy task. This test also allows to calculate the Central Coherence Index (CCI), ranging from 0 (detailed) to 2 (global), which comes out from the order of the construction index (drawing of global or local elements in the first stage of the copy task) and the style index (the degree of continuity in the drawing process).

Moreover, all patients received periodic general medical evaluations by carrying out blood tests (i.e., blood count, prolactin, liver function, blood sugar, thyroid function), cardiological examination, according to good clinical practices.

### 2.2. Statistical Analysis

Data were analyzed using the Statistical Package for Social Sciences Version 26 (SPSS, Chicago, IL, USA). Descriptive statistics included frequencies and percentages, means, and standard deviations, as appropriate. Differences between groups were explored through chi-squared and t-tests, as appropriate.

The general Linear Model (GLM)—Repeated Measures test was applied to determine differences over time of assessments along 15 months (t_0_: baseline; t_1_: 3 months; t_2_: 12 months; t_3_: 15 months) and tested differences between drugs. Specifically, we conducted a head-to-head comparison between the Ari-LAI and PP1M group that completed the 15-month follow-up observation. Then, we performed an evaluation between patients who continued PP1M for the full 15 month and patients who switched to PP3M in the last 3 months of the study. The level of statistical significance was set at *p* < 0.05.

## 3. Results

At the outset, a group of 69 patients were screened for the study. However, 23 of these individuals were excluded from the study for a variety of reasons. Among these, 14 patients either lacked interest in participating or were illiterate, while nine participants withdrew from the study before treatment completion. Specifically, five patients in the Ari-LAI group and four in the PP1M group dropped out. Of the Ari-LAI group, two patients dropped out due to lack of clinical response, two due to non-adherence, and one due to relocation. In contrast, all dropouts in the PP1M group were due to non-adherence. Consequently, the final sample comprised 46 participants, who were consecutively recruited for this 15-month real-life clinical study.

**Figure 2:**
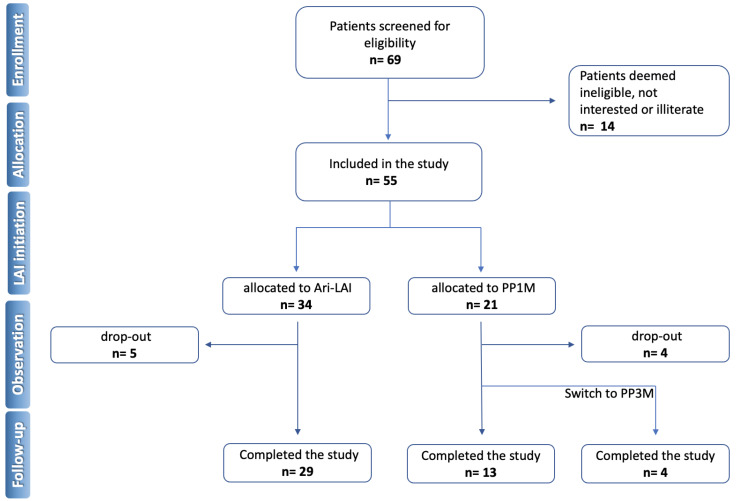
Study flow-chart. Ari-LAI: LAI aripiprazole; LAI: Lon-acting injectable; PP1M: paliperidone palmitate 1 month; PP3M: paliperidone palmitate 3 months.

Participants were divided into three groups according to clinical judgment: Group 1 (29 patients treated with Ari-LAI), Group 2 (13 patients treated with (PP1M), and Group 3 (four patients treated with PP3M). Additionally, the low patients dropout rate (i.e., 16.4%), with complete response rate over 83.6% denotes a high acceptance of the treatment and assessment tools by the patients (Figure 2).

We did not identify any relevant group differences in the demographic composition of the groups (i.e., age, smoking habits, years of education, marital status, employment status, oral treatment, hospitalizations, and duration of illness). Furthermore, Table 1 displays the t_0_ group differences in psychopathological, functional, and neuropsychological variables. There were no baseline group differences in any of the administered tests (Table 1).

The GLM Repeated Measures results about assessment over time and between groups are reported in Table 2. These findings suggest a positive effect of time on scores of PANSS Total, Positive, Negative, and General, QoLS, PSP, Stroop test, RCFT Recall and OS, regardless of the type of SGA-LAI used.

Table 3 reports GLM repeated measures of the clinical and metabolic variables. The results show a trend towards improvement in both triglycerides and prolactin values over time, although neither reaches full statistical significance (i.e., *p* = 0.079 and *p* = 0.067). Total cholesterol levels, on the other hand, show a statistically significant improvement over time (*p* = 0.003, ƞ^2^ = 0.331). Finally, the values of neutrophils, platelets, and TSH remain stable over time and with respect to the type of LAI used, without undergoing any particular variations in terms of average reduction or increase.

Finally, Table 4 reports GLM repeated measures results of PANSS and PSP scales among patients who continued on PP1M and PP3M. We found several significant results in the switch from PP1M to PP3M regarding PANSS total, negative, and general, and PSP, while PANSS positive scale showed an improvement trend as well.

## 4. Discussion

This prospective, naturalistic, independent study attempted to evaluate the effectiveness of SGA-LAIs after switching from oral formulations in a real-world sample of patients suffering from SCZ, with a 15-month follow-up time. Our findings reported significant improvement in all explored domains, including psychopathological, cognitive, functional, and general health, thus confirming with a larger sample and longer observation time our previous conclusions [16].

The clearest result of our study is the positive effect of LAI therapy on several investigated variables over time, rather than being linked to the type of molecule chosen, with findings that seem to strengthen as the evaluation lengthens. This speculation is supported by literature data on treatment management, which underline how APs represent a cornerstone in the treatment of SCZ, but their effectiveness is also linked to the constancy of their intake, as demonstrated by long-term trials [24,25]. Hence the concept of “time as a drug,” according to which the potential benefit of LAI medications is maintained or even improved in the long term both in clinical, general health, and rehabilitation terms, especially when introduced in early phases of the disease [26,27,28,29,30].

Considering the observational nature of our study, the clinical choice for all patients was to continue the administration of LAI therapy at the same dose, in light of the good tolerability and excellent efficacy observed. This clinical choice led to significant improvement in psychopathological, cognitive, social, and clinical domains, in the absence of relapses, and it was supported by robust evidence [31,32]. In fact, a recent network meta-analysis explored the possibility of continuing, reducing, switching, or stopping APs medications in clinically stable patients suffering from schizophrenia-spectrum disorders, concluding that maintaining or switching AP therapy, including the LAI formulation, reduces the risk of clinical relapse with respect to dose interruption or reduction [33].

The second most relevant result of this work is that we did not identify significant differences in tolerability and effectiveness profiles between the Ari-LAI and the PP1M/PP3M groups. While, on the one hand, this could be explained by the reduced sample size, it should, however, be considered that this data remains in continuity with both what was assessed in our previous study [16] and similar researches [34,35]. Indeed, a retrospective study in a real-world clinical setting comparing the effectiveness of 1 year treatment with Ari-LAI, haloperidol LAI and PP1M in a sample of patients with SCZ identified similar tolerability and effectiveness profiles [36]. Thus, it may be deduced once again that the timely initiation of therapy and the maintenance of the peculiar LAI pharmacokinetics are more important than the choice of the molecule itself [36,37].

When considering global tolerability and metabolic parameters, Ari-LAI and PP1M showed similar effects on dropout rate and weight gain [38,39] as we found. However, contrary to our findings, Ari-LAI aripiprazole seems to be associated with lower triglycerides and prolactin blood levels [39]. Overall, it is interesting to note that both groups reported an improvement in triglycerides, cholesterol, and prolactin levels, although the only significant result was related to total cholesterol over time, with no group difference. This finding is in line with similar evidence suggesting that switching from an oral AP to LAI formulation may lead to improvements in cholesterol and prolactin levels in individuals with SCZ [11,40]. This effect is particularly evident considering both the greater therapeutic adherence and the feasible general improvement in lifestyle, diet, and physical activity consequent to functional recovery [41,42,43], as well as the potential disease-modifying effect on symptoms, functional outcome, and mortality [44,45]. However, considering that the results of studies in this area have been mixed, more research is needed to fully understand the effects of such a switch on metabolic parameters [11].

The novelty lies in the lack of significant impact on neutrophils and platelets, which has not been extensively studied in the current literature. While some existing data on the correlation between APs and white blood cells exist, there is limited information on the effects of APs on platelets. Prior research has shown that short-term AP treatment leads to a decrease in total white blood cell count, while long-term treatment leads to an increase [46]. Thus, based on this speculation, a lower total white blood cell count may be indicative of a better response to APs, and our prospective study can aid in elucidating this aspect.

Finally, we found some interesting findings in the subgroups of patients switching to PP3M after 12 months of constant administrations of PP1M. Clearly, the limited data on only four patients prevent us from drawing any conclusions, but they certainly bode well for a broader and more in-depth prospective evaluation. Nevertheless, there is literature in support of a substantial benefit of the switch from PP1M to PP3M with respect to numerous parameters, although a global evaluation of psychopathological, cognitive, social, and clinical domains is still lacking [47]. Recently, a 3 month prospective study investigating the effect on prolactin, sexual function, and clinical symptomatology of switching to PP3M identified a significant benefit in a group of 25 patients affected by SCZ, similar to what has been suggested by our sample [48]. Likewise, the switch to the quarterly formulation of paliperidone palmitate demonstrated the maintenance of clinical stability, a reduced number of hospitalizations, and improvement in clinical symptomatology, with a probable positive effect also in terms of tolerability, while solid data in terms of cognition and quality of life are lacking [48,49,50].

The cognitive assessment, while reporting a globally positive or non-worsening trend over time, did not identify significant results, except for the Stroop test and the percentage of recall and organizational strategies in the RCFT, thus confirming our previous results [16]. This improvement in assessment correlates with better patient performance in cognitive flexibility, interference control, verbal speed, and attention domains, as well as visual organization, with practical implication in everyday life [51,52].

The findings described in the present study should be interpreted with caution considering some methodological limitations. First, the relatively small sample size does not allow generalization of our results to a broader clinical population. This is particularly noticeable for some variables such as general health or quality of life domains, where studies with larger samples have achieved more solid results [53]. Thus, to improve data accuracy, increasing the sample size in future similar prospective research would be desirable. Second, we recognize that our study’s naturalistic design inherently includes some disparities in clinical severity at baseline (i.e., t_0_ mean PANSS global score) and differences in the prescribed dosage in the groups (i.e., Ari-LAI 300–400 mg/month, PP1M 100–150 mg/4 weeks, PP3M 350–525 mg/12 weeks), although these differences are not statistically significant. Indeed, we consider that the limitations stated above may also be considered peculiar strengths, considering that we conducted a real-world and non-randomized controlled study allowing the evaluation of the real-life SGA-LAIs outcomes on patients, whereas the structured approach of a clinical trial may not reflect what happens in routine clinical practice [54]. Improvement in clinical, functional, and neuropsychological assessment in research does not necessarily translate to the effectiveness in clinical practice parameters, such as employment or work readiness. On this regard, the randomized and head-to-head QUALIFY study compared the relationship between validated measures of quality of life, functioning, and tolerability and readiness to work, concluding that the improvement in the clinical domains can also be expressed in a real improvement in enhanced work readiness if properly explored [55].

The last limitation regards the assessment, in fact, it would be appropriate to corroborate our findings with future studies involving the use of larger neuropsychological batteries with more attention to other variables such as cognitive reserve or using the MATRICS Consensus Cognitive Battery (MCCB), considered the golden standard to evaluate cognitive functions in SCZ [56]. On the other hand, the choice to use more feasible tests falls within the desire to make the methodology easily replicable in everyday clinical contexts and settings.

## 5. Conclusions

The switch from oral antipsychotic therapy to SGA-LAI represents a valid and effective treatment strategy, also in terms of safety and tolerability, for patients suffering from schizophrenia.

Our data demonstrate that the benefit is maintained over time, regardless of the type of SGA-LAI chosen (i.e., aripiprazole monohydrate, paliperidone palmitate) or the duration of the formulation (i.e., PP1M, PP3M). Indeed, patients treated continuously with SGA-LAI reported excellent therapy adherence and good tolerability, as demonstrated by the low study dropout rate, with significant improvements on psychopathological, cognitive, social, and metabolic variables over time.

The lack of differences in response between Ari-LAI, PP1M, and PP3M in this study with a naturalistic and prospective design implies that timely initiation and subsequent continuity of LAI therapy, in patients who have already benefited from oral treatment, may be more important than the choice of the type of SGA-LAI itself.

Our findings suggest that shifting to SGA-LAI treatment may lead to a favorable outcome on functional recovery besides full clinical remission, thus facilitating the success of long-term psychosocial interventions in SCZ.

## Figures and Tables

**Table 1:** Baseline demographics and assessment of the sample.

	Ari-LAI (*n* = 29)		PP1M (*n* = 17)		Statistics	*p*
Age ^a^	42.5	15.3	43.1	13.7	*t* = 0.141	0.888
Sex ^b^					χ^2^ = 3.490	0.062
Male	16	55.2	14	82.4		
Female	13	44.8	3	17.6		
Smoker (Yes) ^b^	13	44.8	12	70.6	χ^2^ = 2.867	0.090
Years of Education ^a^	11.1	3.2	12.2	3.5	*t* = 1.153	0.255
Marital Status ^b^					χ^2^ = 2.646	0.450
Single	20	69	13	76.4		
Married	7	24.1	2	11.8		
Divorced	2	6.9	2	11.8		
Employment ^b^					χ^2^ = 2.714	0.744
Self-employment	4	13.8	2	11.8		
Employed	6	20.7	3	17.6		
Unemployed	12	41.4	4	23.5		
Disabled	5	17.2	6	35.3		
Student	2	6.9	2	11.8		
Oral Treatment ^b^						
Antidepressants	13	44.8	4	23.5	χ^2^ = 2.087	0.149
Antipsychotics	5	17.2	2	11.7	χ^2^ = 0.249	0.618
Benzodiazepines	9	31.0	8	47.1	χ^2^ = 0.712	0.399
Mood Stabilizers	9	31.0	8	47.1	χ^2^ = 1.181	0.277
Patients with previous hospitalizations ^b^	16	55.2	9	52.9	χ^2^ = 3.641	0.725
Duration of illness (years) ^a^	15.3	8.1	12.5	6.5	*t* = 1.343	0.242
PANSS ^a^						
Total	84.5	16.9	96.6	22.5	*t* = 0.558	0.580
Positive	18.1	7.6	19.0	5.2	*t* = −0.221	0.826
Negative	21.6	6.9	26.0	9.8	*t* = 1.140	0.260
General	44.7	7.6	51.2	11.6	*t* = 0.399	0.692
QoLS ^a^	67.5	15.5	61.3	18.7	*t* = −0.145	0.886
PSP ^a^	53.2	11.7	48.4	16.5	*t* = −0.435	0.665
Stroop ^a^	−2.1	6.9	−3.2	8.4	*t* = 1.268	0.212
RCFT ^a^						
Accuracy	32.1	6.0	30.9	3.8	*t* = 2.136	0.083
Percentage of Recall	51.8	15.5	56.3	17.2	*t* = −1.173	0.247
Order	1.8	0.9	1.5	0.9	*t* = 0.317	0.753
Style	1.5	0.5	1.5	.5	*t* = 0.464	0.645
CCI	1.3	0.5	1.3	0.4	*t* = 0.913	0.366
OS	4.7	2.0	3.8	1.5	*t* = 1.509	0.139

Ari-LAI: LAI aripiprazole; CCI: Central Coherence Index; LAI: Lon-acting injectable; OS: Organizational Strategies; PANSS: Positive and Negative Syndrome Scale; PP1M: paliperidone palmitate 1 month; PP3M: paliperidone palmitate 3 months; PSP: Personal and Social Performance Scale; QOLS: Quality of life scale; RCFT: Rey-Osterrieth Complex Figure Test. ^a^ Results are presented as means (SD). ^b^ Results are presented as frequencies (%).

**Table 2:** General linear model repeated measures of the assessment.

Scale	Subscale	Groups	t_0_	t_1_	t_2_	t_3_	F	*p*	ƞ^2 a^
PANSS Total	Total	Ari-LAI	84.5	72.1	65.2	67.1	Time: F = 10.990	**<0.001**	0.622
		PP1M	96.6	74.1	71.3	70.1	Group: F = 0.821	0.498	
	Positive	Ari-LAI	18.1	14.9	13.2	14.2	Time: F = 3.712	**0.028**	0.358
		PP1M	19	14.1	12.6	13.7	Group: F = 0.159	0.923	
	Negative	Ari-LAI	21.6	19.4	17.7	18.2	Time: F = 5.928	**0.005**	0.471
		PP1M	26	20.1	20.2	21	Group: F = 0.768	0.525	
	General	Ari-LAI	44.7	38.3	34.5	34.7	Time: F = 13.280	**<0.001**	0.667 777
		PP1M	51.2	39.4	38.6	35.7	Group: F = 1.303	0.301	
QoLS		Ari-LAI	67.5	79.12	80.0	81.37	Time: F = 5.856	**0.016**	0.422
		PP1M	61.3	70.45	76.2	77.40	Group: F = 1.19	0.307	
PSP		Ari-LAI	53.2	62.1	63.4	65.9	Time: F = 9.530	**<0.001**	0.588
		PP1M	48.4	58.4	61.4	60.6	Group: F = 0.359	0.783	
Stroop		Ari-LAI	−2.1	−1.5	2.2	2.6	Time: F = 4.120	**0.002**	0.382
		PP1M	−3.2	−0.6	4.8	−1	Group: F = 0.572	0.064	
RCFT Accuracy	Accuracy	Ari-LAI	32.1	34.4	34.1	33.9	Time: F = 1.271	0.311	
		PP1M	30.9	31.8	33.8	32.9	Group: F = 0.599	0.145	
	Percentage of Recall	Ari-LAI	51.8	65.3	72.4	68.8	Time: F = 4.968	**0.001**	0.427
		PP1M	56.3	53.8	61.4	56.2xf	Group: F = 0.599	0.623	
	Order	Ari-LAI	1.8	2.4	2.2	2	Time: F = 2.225	0.117	
		PP1M	1.5	1.6	1.4	1.8	Group: F = 1.212	0.331	
	Style	Ari-LAI	1.5	1.7	1.8	1.7	Time: F = 1.190	0.339	
		PP1M	1.5	1.4	1.4	1.6	Group: F = 1.842	0.172	
	CCI	Ari-LAI	1.3	1.6	1.6	1.5	Time: F = 1.046	0.394	
		PP1M	1.3	1.2	1.2	1.3	Group: F = 1.556	0.231	
	OS	Ari-LAI	4.7	5	4.9	5.5	Time: F = 6.367	**0.003**	0.489
		PP1M	3.8	4.4	4.4	5.7	Group: F = 0.918	0.450	

Ari-LAI: LAI aripiprazole; CCI: Central Coherence Index; LAI: Long-acting injectable; OS: Organizational Strategies; PANSS: Positive and Negative Syndrome Scale; PSP: Personal and Social Performance Scale; QoLS: Quality of life scale; RCFT: Rey-Osterrieth Complex Figure Test. ^a^ Effect size is calculated for significant results, reported in bold.

**Table 3:** General Linear Model repeated measures of the metabolic variables.

Variable	Groups	t_0_	t_3_	F	*p*	ƞ^2 a^
Triglycerides (mg/dL)	Ari-LAI	160	146	Time: F = 3.394	0.079	
	PP1M	147	130	Group: F = 0.062	0.806	
Total cholesterol (mg/dL)	Ari-LAI	191	174	Time: F = 11.375	**0.003**	0.331
	PP1M	183	163	Group: F = 0.112	0.74	
Prolactin (ng/mL)	Ari-LAI	17.6	10.4	Time: F = 3.827	0.067	
	PP1M	41.1	34.2	Group: F = 0.03	0.959	
Neutrophils (number 10^3^/mm^3^)	Ari-LAI	5.8	5.9	Time: F = 0.02	0.964	
	PP1M	4.9	4.8	Group: F = 0.252	0.62	
Platelets (number 10^3^/mm^3^)	Ari-LAI	272	270	Time: F = 0.36	0.852	
	PP1M	240	245	Group: F = 0.220	0.643	
TSH (µU/mL)	Ari-LAI	2.3	2.1	Time: F = 1.844	0.189	
	PP1M	2.2	2.1	Group: F = 0.380	0.544	

Ari-LAI: LAI aripiprazole; dL: deciliter; mg: milligrams; ng: nanogram; LAI: Lon-acting injectable; ml: milliliter; mm: millimeter; PP1M: paliperidone palmitate 1 month; TSH: thyroid stimulating hormone; μU: microunit. ^a^ Effect size is calculated for significant results, reported in bold.

**Table 4:** General Linear Model repeated measures of assessments and clinical variables between PP1M and PP3M.

Scale	Subscale	Groups	t_2_	t_3_	F	*p*	ƞ^2^
PANSS	Total	PP1M > PP3M	65	51	Time: F = 26.133	**0.01**	0.897
	Positive	PP1M > PP3M	13	10	Time: F = 8.593	0.061	0.741
	Negative	PP1M > PP3M	21	15	Time: F = 10.475	**0.05**	0.777
	General	PP1M > PP3M	32	27	Time: F = 13.636	**0.03**	0.82
PSP		PP1M > PP3M	59	73	Time: F = 30.351	**0.01**	0.91
Triglycerides (mg/dL)		PP1M > PP3M	155	135	Time: F = 19.469	**0.022**	0.866
Total cholesterol (mg/dL)		PP1M > PP3M	190	176	Time: F = 3.319	0.166	0.525
Prolactin (ng/mL)		PP1M > PP3M	34.3	27.2	Time: F = 8.610	0.06	0.742
Neutrophils (number 10^3^/mm^3^)		PP1M > PP3M	5.2	5.2	Time: F = 0.009	0.932	0.003
Platelets (number 10^3^/mm^3^)		PP1M > PP3M	274	280	Time: F = 0.549	0.513	0.155
TSH (µU/mL)		PP1M > PP3M	2.22	2.07	Time: F = 14.575	**0.032**	0.829

dL: deciliter; mg: milligrams; ml: milliliter; mm: millimeter; ng: nanogram; PANSS: Positive and Negative Syndrome Scale; PP1M: paliperidone palmitate 1 month; PP3M: paliperidone palmitate 3 months; PSP: Personal and Social Performance Scale; TSH: thyroid stimulating hormone; μU: microunit. Significant results are reported in bold.

## Data Availability

The data that support the findings of this study are available from the corresponding author upon reasonable request.
